# Nanotechnology-Based Nucleic Acid Vaccines for Treatment of Ovarian Cancer

**DOI:** 10.1007/s11095-022-03434-4

**Published:** 2022-11-14

**Authors:** Simav Gildiz, Tamara Minko

**Affiliations:** 1 Department of Pharmaceutics, Rutgers, the State University of New Jersey, Piscataway, NJ USA; 2 Rutgers Cancer Institute of New Jersey, Rutgers, the State University of New Jersey, New Brunswick, NJ USA

**Keywords:** cancer, mRNA, nanoparticles, ovarian, vaccine

## Abstract

Anticancer vaccines represent a promising approach for effective treatment of cancer and along with recent advantages of nucleic acid-based vaccines for other diseases form a prospective and potentially efficacious direction of the research, development and clinical applications. Despite the ongoing several clinical trials of mRNA vaccines for the treatment of various types of cancer, to-date no cancer vaccines were approved by the US Food and Drug Administration. The present review analyzes and summarizes major approaches for treating of different forms of ovarian cancer including mRNA-based vaccines as well as nanotechnology-based approaches for their delivery.

**Graphical Abstract:**

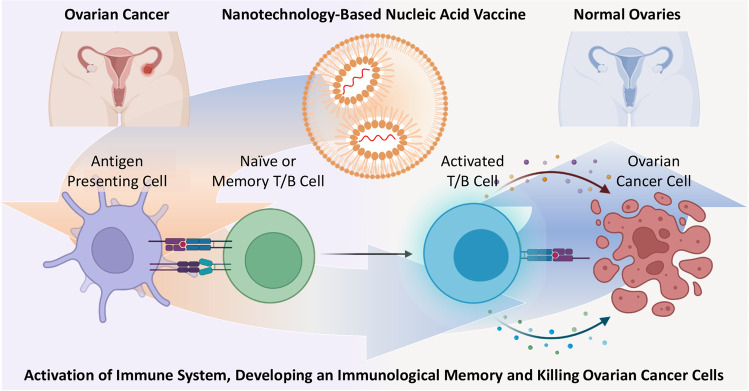

## Introduction

Ovarian cancer ranks fifth in the cancer deaths of women and is the leading cause of death from gynecologic cancers in developed countries [1, 2]. It was previously believed that ovarian cancer can be initiated only within the ovary. However, recent data show that morphologically ovarian tumors usually represent heterogeneous neoplasms with differing molecular fingerprints and clinical pathological features and shows little phenotypic similarity with ovarian cell [3]. Approximately 2.7% women have a high chance of developing ovarian cancer during their life when all protective measures are absent [4]. It is estimated that that around 20,000 women will be diagnosed with ovarian cancer in the US and more than 12,000 deaths from ovarian cancer are expected [1]. Globally, 313,959 new cases have been diagnosed and 207,252 women have died from ovarian cancer in 2020 [5]. Late diagnosis and a high relapse after first line of therapy usually cause high mortality of patients with ovarian cancer [3, 6]. The majority (75%) of women are diagnosed at the stage III of ovarian cancer when metastatic disease already spread to the peritoneal cavity indicating a poorer prognosis [3, 7, 8]. The late diagnosis presents a major obstacle in the treatment of ovarian cancer leading to the steep decline in the survival rate from 89% within 5 years in stage I cancers to 41% in advanced stages [9]. In the present work, we review the origin, risk factors, diagnosis and treatment options of ovarian cancer. Also, cancer vaccines in clinical trials, as well as perspectives of development and methods of nanotechnology-based delivery of mRNA-based vaccines are discussed.

## Forms of Ovarian Cancer

**Fig. 1:**
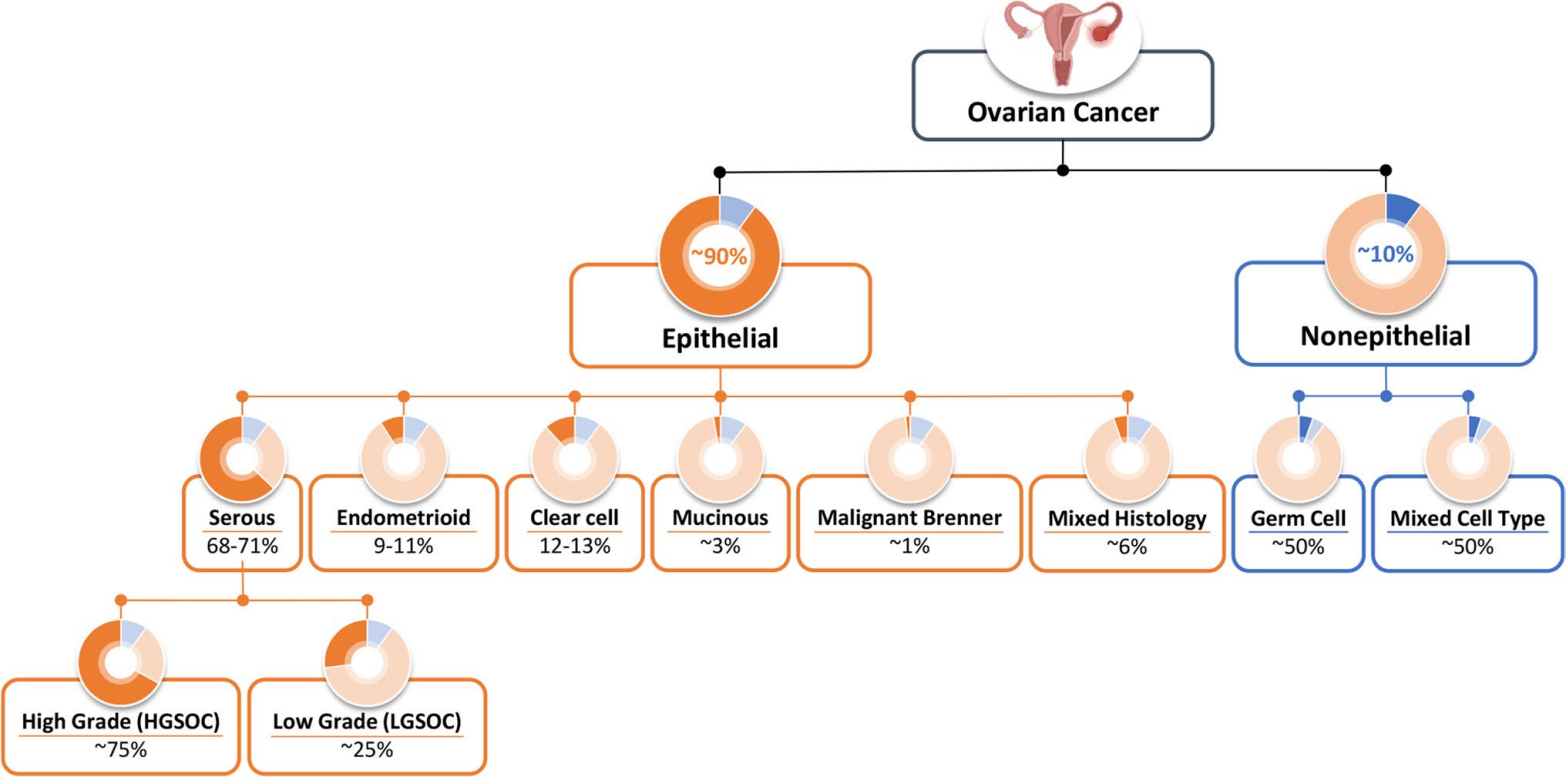
Types of Ovarian Cancer. Created based on data from [2, 13].

Ovarian cancer can be classified in differing subtypes. About 90% of all ovarian malignancies have originated from epithelial cells [2, 10–12] (Fig. 1). Only about 5% of malignant ovarian tumors have germ cell and sex-cord stromal origin while the rest has a mixed cell phenotype [2, 13]. In turn, primary epithelial cancer usually is subdivided based on cell histology on four major subtypes: serous, endometrioid, mucinous, and clear cell carcinomas (Fig. 1) [2, 13, 14]. Serous tumors are categorized into two subtypes: high grade serous carcinoma (HGSC) and low-grade serous carcinoma (LGSC) [13, 15, 16]. From 70 to 80% of all subtypes of epithelial ovarian cancer are HGSCs, while LGSCs account for less than 5% [13].

### Epithelial Ovarian Cancers

From histologic, molecular and cytogenetic points of view, ovarian cancer is a diverse disease. As mentioned above, there are multiple histological subtypes of this malignancy dividing it into four primary categories. In addition to these subtypes, malignant Brenner tumors and mixed subtypes have a serous histology [2]. Serous carcinomas make up about 70% of all epithelial ovarian cancer, following with clear cell carcinoma accounting for 12–13%, endometrioid carcinomas accounting for 9–11% and mucinous carcinomas accounting for only 3% of epithelial ovarian cancers. Epithelial ovarian cancer malignancies can also be subdivided into two categories: type I and type II malignancies [13, 15, 17, 18]. Type I malignancies tend to be large cystic neoplasms detected at low grade stage, whereas type II malignancies are almost always diagnosed at an advanced stage [19]. Type I malignancies are low in proliferative activity and progress slow and indolent [19]. Type II malignancies on the other hand progress fast and aggressively and have high proliferative activity [19]. They also have differing origin sites and genetic mutations in the cancer cells as shown in Table I.

**Table I:** Origin, Which Genetic Mutations are Found, and the Cancer Types Included in Type I and Type II Malignancies in Epithelial Ovarian Cancer. Modified from [2]

Cancer types	Origin	Genetic mutations	Cancer types
Type I	Mostly arises from endometriosis, ovaries or fallopian tubal-related serous borderline ovarian tumors	KRAS BRAF PTEN PIK3CA CTNNB1 ARID1A	LGSC Endometrioid carcinoma Clear-cell carcinoma Mucinous carcinoma Malignant Brenner tumor Seromucinous carcinoma
Type II	Originates in the fallopian tube epithelium	TP53	HGSC Undifferentiated carcinoma Carcinosarcoma

#### Serous Carcinomas

HGSC and LGSC account for the majority of all epithelial ovarian cancer; however, there are still substantial differences between the two. The major difference is in the malignancy types of HGSC and LGSC. HGSC is the most common type of serous ovarian cancer and accounts for more than 90% of all serous ovarian cancers [13]. This is the most aggressive form of ovarian cancer and belongs to type II malignancy [2, 20]. It is also the deadliest form of epithelial ovarian cancer as the 5-year survival rates are 29% and 13% for late stage II and IV, respectively [21, 22]. HGSC can arise from ovaries, the fallopian tube or the peritoneum, but it is hard to precisely determine the origin as it most often presents in an advanced form where the disease has metastasized [20, 23, 24].

LGSC, on the other hand, is a type I malignancy which has different precursors and molecular pathways when compared with HGSC [21]. These carcinomas account for less than 10% of the serous carcinomas and are usually detected at a stage where the disease is confined to the ovary [25, 26]. They are slow progressing in nature due to the type I malignancy and have better prognosis compared to HGSC [13, 25]. Women are also diagnosed at younger ages with LGSC [13]. The origin of LGSC is believed to begin in the ovaries.

#### Endometrioid Carcinomas

Endometriosis and endometrioid carcinomas have been associated for the origin of this subtype of cancer [27–30]. It is hypothesized that endometrioid carcinomas originate from endometriosis and are usually detected at earlier stages [13, 31]. An earlier diagnosis results in better prognosis and survival, but the histology is also chemotherapy sensitive making the treatment more successful [13].

#### Clear Cell Carcinomas

Clear cell carcinomas (CCC) are the second most common type of epithelial ovarian cancer [32]. CCC as well as endometrioid cancers have relatively good prognosis due to diagnosis at early stages [13]. When compared to HGSC, CCC usually present at lower stages of the disease and are typically considered a type I malignancy. However, if they are diagnosed at a later stage, the prognosis gets worse as they are more resistant to chemotherapy with platinum or taxanes [21, 33–35].

#### Mucinous Carcinomas

Mucinous carcinomas are one of the rarest types of epithelial ovarian cancer and they only make up about 1–3% of the cases and are often diagnosed as borderline tumors or at stage I [13, 36]. This subtype usually has a good prognosis due to early detection [21].

#### Germ Cell Carcinomas

Germ cell carcinomas account for only 3% of all ovarian cancer cases and are considered rare [13]. They are usually diagnosed at a young age, with an average age between 10–30 years [13, 37, 38]. This type of cancer is known to be producing specific type of tumor markers which helps in their diagnosis and the selection of a treatment plan [13, 39, 40].

#### Sex-cord Stromal Carcinomas

Sex-cord stromal carcinomas are the rarest ovarian neoplasms and are usually not malignant [13, 41]. The subtype is usually diagnosed very early and are more common in African American women than white women [13, 41].

## Risk Factors

Numerous risk factors are associated with developing ovarian cancer. On the other hand, clinical correlations give us the ability to select some aspects that could have a protective effect against the development of ovarian cancer (Table II).

**Table II:** Predisposing and Protective Factors Related to Developing Ovarian Cancer in Women. Modified from [12]

Factors	Predisposing	Protective
	X	
Age	X	
Pregnancy		X
Age at childbirth		X
Endometriosis	X	
Use of contraception	X	
Breastfeeding		X
Family history	X	
BRCA mutation	X	
Lynch syndrome	X	

Epithelial ovarian cancer is considered to be a post-menopausal disease and is age related [12, 41, 42]. Women over the age of 65 are in a higher risk of developing epithelial ovarian cancer and the median age at diagnosis is 50–79 years [12, 42–45]. Older age is also associated with lower survival rate when compared to younger age [12, 41]. However, this can also be due to the use of less aggressive therapies with older women resulting in lower survival [12, 46]. Unfortunately, older age (> 64 years) is a predictor of high mortality in patients with ovarian cancer [12, 47].

In addition to demographic related factors, reproductive factors also play a role in the development of ovarian cancer. There is some evidence showing that non-mucinous ovarian cancers are associated with a development of menstrual periods and ovulation cycles, which is reasonable considering the constant change of epithelial tissue related to these events [12, 48]. The relationship between the risk of developing ovarian cancer and having ovulation cycles is inverse in nature and numerous studies have showed this relationship [12]. Related to this association, pregnancy has a protective role over the development of ovarian cancer as many studies have shown [12, 49–54]. A case study has shown that women who have given birth or had an induced abortion were less likely to develop ovarian cancer, and the risk of developing ovarian cancer decreased with each live birth [12]. Another factor related to this issue is the age at childbirth. The older the age at childbirth, the less risk of developing this disease have been shown in previous studies [12, 50].

As mentioned before, having endometriosis is a predisposition to developing endometrioid cancer. As early as 1925, the link between endometriosis progressing into endometrioid carcinoma has been demonstrated [12, 55]. Hormonal factors on the other hand could be a protective measure against ovarian cancer. Using oral contraceptives has been shown to decrease the risk of developing all types of ovarian cancer in numerous studies [12, 48, 54, 56, 57]. The influence of these factors could be related to the suppression of ovulation cycles. Interestingly, breastfeeding can also have a protective effect against ovarian cancer. Previous studies have found an inverse relationship between the duration of breastfeeding, the number of children that were breastfed and risk of having ovarian cancer [12, 53, 58]. It should be however stressed, that the correlation between two parameters does not automatically mean that one of such variables is a direct cause of another. In fact, they both may be a result of some unknown third (or even more) cause(s).

Lastly, genetics plays a substantial role in the development of ovarian cancer. The three main genetic risk factors include a family history of breast or ovarian cancer, BRCA1 and BRCA2 mutations, and lynch syndrome [12]. Having family history of breast or ovarian cancer is the main risk factor for developing this disease, and a personal history of breast cancer also increases the risk of developing ovarian cancer [9, 12, 59, 60]. Around 14% of epithelial ovarian cancers are due to BRCA1 and BRCA2 mutations, and about 65–85% of inherited ovarian tumors have resulted from a mutation in the BRCA germline [10, 12, 61]. Hereditary non-polyposis colorectal cancer, also known as Lynch syndrome, is an autosomal dominant cancer predisposition syndrome that is the most common cause for hereditary colon cancer and is responsible for 1–3% of all colorectal cancer [12, 62, 63]. It was shown that around 10–15% of the total inherited ovarian cancer cases is due to the Lynch syndrome [12, 64].

## Molecular Characteristics of Ovarian Cancer

**Fig. 2:**
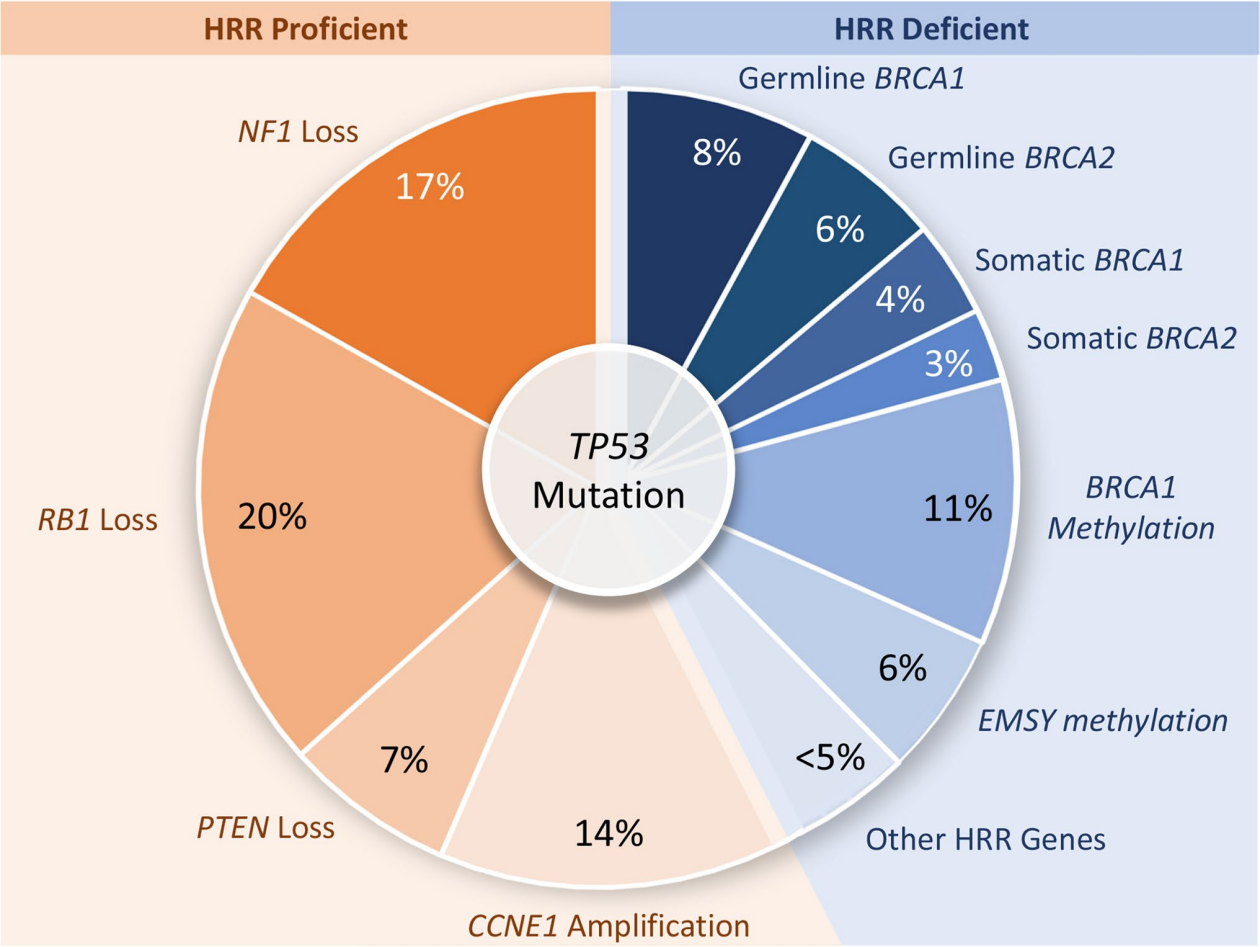
Mutations in high grade serous carcinoma (HGSC) related to homologous recombination DNA repair (HRR) pathway. Redrawn from [65].

It was found that certain genetic mutations are common within patients with HGSC, the most ubiquitous one being *TP53* mutations [65–68]. This gene encodes a tumor suppressor protein, p53, and it is lost through missense, frameshift or nonsense mutations [65, 69]. One differentiating feature of mutations in HGSC when compared to other tumor types is more chromosomal instability and defective repair of DNA instead of activation of oncogenes [65, 70, 71]. Almost 50% of HGSC mutations are in genes related to the homologous recombination repair pathway (HRR), and it includes germline, somatic and epigenetic type of mutations (Fig. 2, [65]). One of the most prominent mutations is in the *BRCA* genes which accounts for more than 20% of all mutations related to HGSC [65, 72]. As can be seen in Fig. 2, around 14% of *BRCA* mutations are germline mutations, whereas somatic mutations account for approximately 7% [65, 73, 74]. *BRCA1* is a key gene for DNA repair, regulation of transcription and control of cell cycle checkpoint; *BRCA2* is also crucial for DNA repair and HRR [15, 70].

Mutations not related to the HRR pathway include loss of *NF1*, *RB1* and *PTEN* and amplification of *CCNE1* genes. *NF1* is a tumor suppressor gene that negatively regulates the Ras signal transduction pathway, and its loss leads to Ras pathway activation and uncontrollable mitosis [75]. *RB1* is also a tumor suppressor gene; and the loss of the encoded protein has been linked to the genomic instability and cancer cell differentiation, survival, and senescence [76–80]. *PTEN* is another tumor suppressor gene that is commonly mutated in a variety of cancers [81]. *CCNE1* on the other hand, is known oncogene which amplification has been considered as a predictive biomarker for chemotherapy resistance in epithelial cancer [82].

## Diagnosis and Screening Techniques

As previously mentioned, the average age of diagnosis of ovarian cancer is 50–79 years with the median age being 63 [83]. Most women show symptoms such as presence of ascites, which is fluid in the peritoneal cavity, and gastrointestinal dysfunction in the form of bowel obstruction, nausea, gastrointestinal reflux, constipation etc. [15]. Abdominal bloating, pain in the abdomen or pelvic area, fatigue and shortness of breath can also be counted as alarming symptoms [15, 84]. Symptoms of ovarian cancer are often missed at the beginning of the disease because they are nonspecific, general and can be attributed to other diseases [15]. Correspondingly, the diagnosis is frequently given at an advanced stage of cancer – typically stage III or IV – when the symptoms have become noticeable and require an intervention [15, 84]. Symptoms at this stage are severe and indicate peritoneal carcinomatosis extensively, presence of ascites and involvement of cancerous spread in the bowel [15].

Diagnostic work up of the patients include a physical examination of patient that involves a pelvic and rectovaginal examination. Additionally, radiographic imaging such as trans vaginal ultrasonography, abdominal ultrasonography, CT, MRI or PET scans are used [15]. Ovarian carcinoma antigen (CA125) serum assay is also used for the detection in the blood [15]. CA125, also known as Mucin16, represents a protein which is produced by the most of epithelial ovarian cancers in their advance stages. Furthermore, to determine the histology of the tumor, laparoscopic surgery can be performed to remove some of the mass [15, 85]. In more advanced stages of the cancer a tumor biopsy is also taken [15]. All these diagnostic tools help to determine the location, size and histological origin of the tumor, to estimate the stage of the cancer and suggest a most promised type of cancer therapy.

**Fig. 3:**
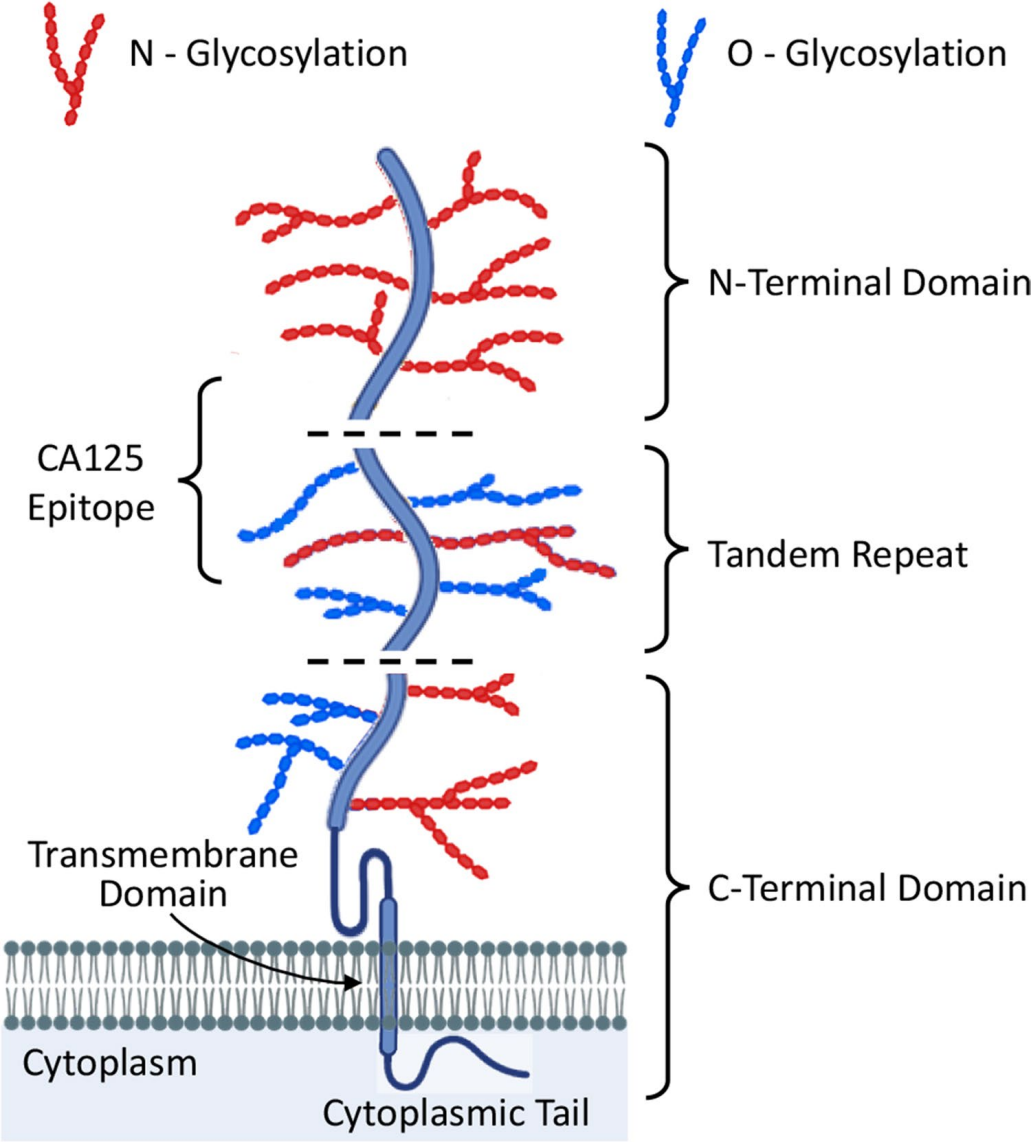
Schematic representation of mucin-16 (CA125) structure: MUC16 contains three domains: the N-terminal domain (~ 12,000 amino acids in length), tandem repeat domain which is interspersed with SEA (sea urchin sperm protein, enterokinase, and agrin) domain and the C-terminal domain. The Tandem Repeat (TR) domain contains 18–60 repeats each with ~ 156 amino acids and has ankyrin (ANK) 1 and 2 sites along with the SEA domains. The C-terminal domain is further divided into an extracellular portion, which contains the putative cleavage site, a transmembrane domain and a cytoplasmic tail of 32 amino acid length. The cytoplasmic tail contains an ERM (ezrin/radixin/moesin) actin-binding domain and a putative nuclear localization signal (RRRKK). Redrawn from [90].

However, as a result of relatively low effectiveness of existing screening techniques, the diagnosis of ovarian cancer takes a long time [15]. Extensive variations in histology and origin of ovarian cancer make the development and validation of effective and universal screening techniques extremely difficult [15]. Among diagnostic assays, CA125 serum test remains an exception and is the most evaluated test for ovarian cancer screening. Increased levels of CA125 are mostly observed in HGSC [15, 86]. It is overexpressed in more than 80% of ovarian cancer cells and nearly undetected by conventional techniques in the normal tissue cells [87, 88]. As can be seen in Fig. 3, Mucin16 (CA125) is a transmembrane mucin with 22,152 amino acids that has a cytoplasmic tail, a single membrane-spanning domain and an N-terminal domain with a tandem repeat sequence (60 + repeats of 156 amino acids) that includes the MUC16 antigen repeat [88–90]. The N-terminal domain of MUC16 has 12,000 amino acids with O-glycosylation only. MUC16 contains about 56 sea-urchin, enterokinase, and agrin (SEA) domains. SEA domain is a common feature among all mucins which is involved in cleavage and association of MUC16 subunits. The transmembrane domain is followed by a 32 amino acids cytoplasmic tail. The location of the CA125 antigen repeat makes it an attractive target for anticancer therapies and the primary object of the mRNA vaccine.

## Staging of Ovarian Cancer

The prognosis and the decision on the type of clinical management for the disease is based on its current stage [91]. Cancer staging is used to describe the anatomical extent of the tumor in the body [92]. Staging in the past relied on clinical examination with limited imagery diagnoses. Now with the advances in imaging techniques such as PET, MRI, CT, ultrasound and surgical staging methods, staging is more defined and usually less invasive [92]. Aims for the classification of clinical staging include help in the planning of treatment and evaluation the results of the treatment. It also facilitates the exchange of information based on patient data between cancer centers or hospitals and creating a large population database on specific malignancies [92, 93].

There are two main systems used for the staging of ovarian cancer: the International Federation of Gynecologists and Obstetricians (FIGO) and the American Joint Committee on Cancer (AJCC) TNM staging system [94]. TNM includes three main parameters: T – the size or direct extent of the primary tumor; N – the degree of spread to regional lymph nodes and M – the presence of distant metastasis. Both systems represent similar characteristics of each stage. Table III shows the stages of ovarian cancer according to FIGO and its diagnostic specifications.

**Table III:** Staging of Ovarian Cancer According to FIGO. Reproduced With Permission from [95]

Stage I: Tumor is confined to one ovary	
IA	Tumor limited to 1 ovary, capsule intact, no tumor on surface, negative washings
IB	Tumor involves both ovaries otherwise like IA
IC:	Tumor limited to 1 or both ovaries
IC1	Surgical spill
IC2	Capsule rupture before surgery or tumor on ovarian surface
IC3	Malignant cells in the ascites or peritoneal washings
Stage II: Tumor involves 1 or both ovaries with pelvic extension (below the pelvic brim) or primary peritoneal cancer	
IIA	Extension and/or implant on uterus and/or Fallopian tubes
IIB	Extension to other pelvic intraperitoneal tissues
Stage III: Tumor involves 1 or both ovaries with cytologically or histologically confirmed spread to the peritoneum outside the pelvis and/or metastasis to the retroperitoneal lymph nodes	
IIIA	IIIA (Positive retroperitoneal lymph nodes and /or microscopic metastasis beyond the pelvis) IIIA1(i) Metastasis ≤ 10 mm IIIA1(ii) Metastasis > 10 mm
IIIA1	Microscopic, extrapelvic (above the brim) peritoneal involvement ± positive retroperitoneal lymph nodes
IIIA2	Microscopic, extrapelvic (above the brim) peritoneal involvement ± positive retroperitoneal lymph nodes
IIIB	Macroscopic, extrapelvic, peritoneal metastasis ≤ 2 cm ± positive retroperitoneal lymph nodes. Includes extension to capsule of liver/spleen
IIIC	Macroscopic, extra-pelvic, peritoneal metastasis > 2 cm ± positive retroperitoneal lymph nodes. Includes extension to capsule of liver/spleen
Stage IV: Distant metastasis excluding peritoneal metastasis	
IVA	Pleural effusion with positive cytology
IVB	Hepatic and/or splenic parenchymal metastasis, metastasis to extra-abdominal organs (including inguinal lymph nodes and lymph nodes outside of the abdominal cavity)

## Treatment of Ovarian Cancer

The primary goal of cancer therapy is to achieve a cure or stop the cancer from progressing and to palliate and minimize the disease symptoms for the patient [15, 96]. Surgery is the main treatment option of ovarian cancer with different type of operations depending on the stage of the cancer [97]. For newly diagnosed patients, primary surgical cytoreduction with a goal of complete resection of the spread carcinoma is usually used [15, 98]. In patients diagnosed with advanced ovarian cancer, maximal cytoreductive surgery has been the key surgical intervention and is an important initial step of the treatment [93]. The principles underlying surgical intervention include the physiological benefit of tumor removal and increased tumor perfusion with adjuvant chemotherapy as well as the increased immunological competence of patient [93].

The use of adjuvant chemotherapy depends on the stage and grade of cancer. Patients with very early-stage cancers, IA or IB, may not need chemotherapy although it can be recommended [16]. In some early cancer stages, observation may be an option after surgery instead of adjuvant therapy [16]. However, chemotherapy may accompany or follow surgical cancer removal. Postoperative use of chemotherapy is recommended in all patients with stage II, III, IV ovarian carcinoma [16]. Patients with a high grade or an advanced stage cancer such as HGSC will undergo platinum-therapy [16, 93]. Combination therapies are also used to maximize the efficacy of the treatment. A combination of platinum-based drugs, taxanes, anti-angiogenic agents, with other drugs is frequently used for the treatment of ovarian cancer [16, 99]. For instance, Bevacizumab (Avastin®)—a humanized anti-VEGF monoclonal IgG1 antibody with molecular weight of 149 kDa, is used as anti-angiogenic agent in combination with carboplatin (platinum-based chemotherapy) and paclitaxel. Such a combination therapy has been approved by the European Medicines Agency as way to enhance the combination therapy [16].

There is an alternative treatment method that has been recently implemented for patients with advanced stage ovarian cancer where the disease is too extensive and a cytoreductive surgery is not possible [100]. The alternative treatment, neoadjuvant chemotherapy (NACT), is the administration of chemotherapy in order to potentially shrink the tumor to make it operable for surgery [100]. Use of NACT has been increasing over past decade as a first line of treatment as clinical trials and survival curves are showing promising results [101]. Clinical studies are still underway in randomized groups with stage III and IV patients that received the traditional treatment with surgery followed by chemotherapy or NACT followed by surgery [102]. An example NACT consists of three cycles of carboplatin and paclitaxel, followed by surgical cytoreduction and finished with an additional three cycles of chemotherapy after the surgery [102].

Unfortunately, almost 70% of advanced stage ovarian cancer patients experience a relapse; even early-stage patients have a 20–25% relapse rate [103]. In order to monitor for recurrent disease, CA125 serum levels are checked after the completion of the treatment plan [104, 105]. However, a level of CA125 in assays substantially varies for different types of ovarian cancer, and therefore it is hard to establish a standard critical level of this protein as an indicator of recurring ovarian cancer [99, 104]. It should also be noted that recurrent ovarian cancer is generally hardly curable unless the tumor is localized, and patient can undergo surgery or radiotherapy. Consequently, other more effective in recurrent ovarian cancer treatment options are required and alternative treatments are emerging [106]. There are studies based on treating platinum resistant recurrent ovarian cancers by using paclitaxel or novel therapeutics such as epothilones, a new agent that inhibits microtubules [104, 106, 107].

### Other Treatment Options and Emerging Therapies

**Fig. 4:**
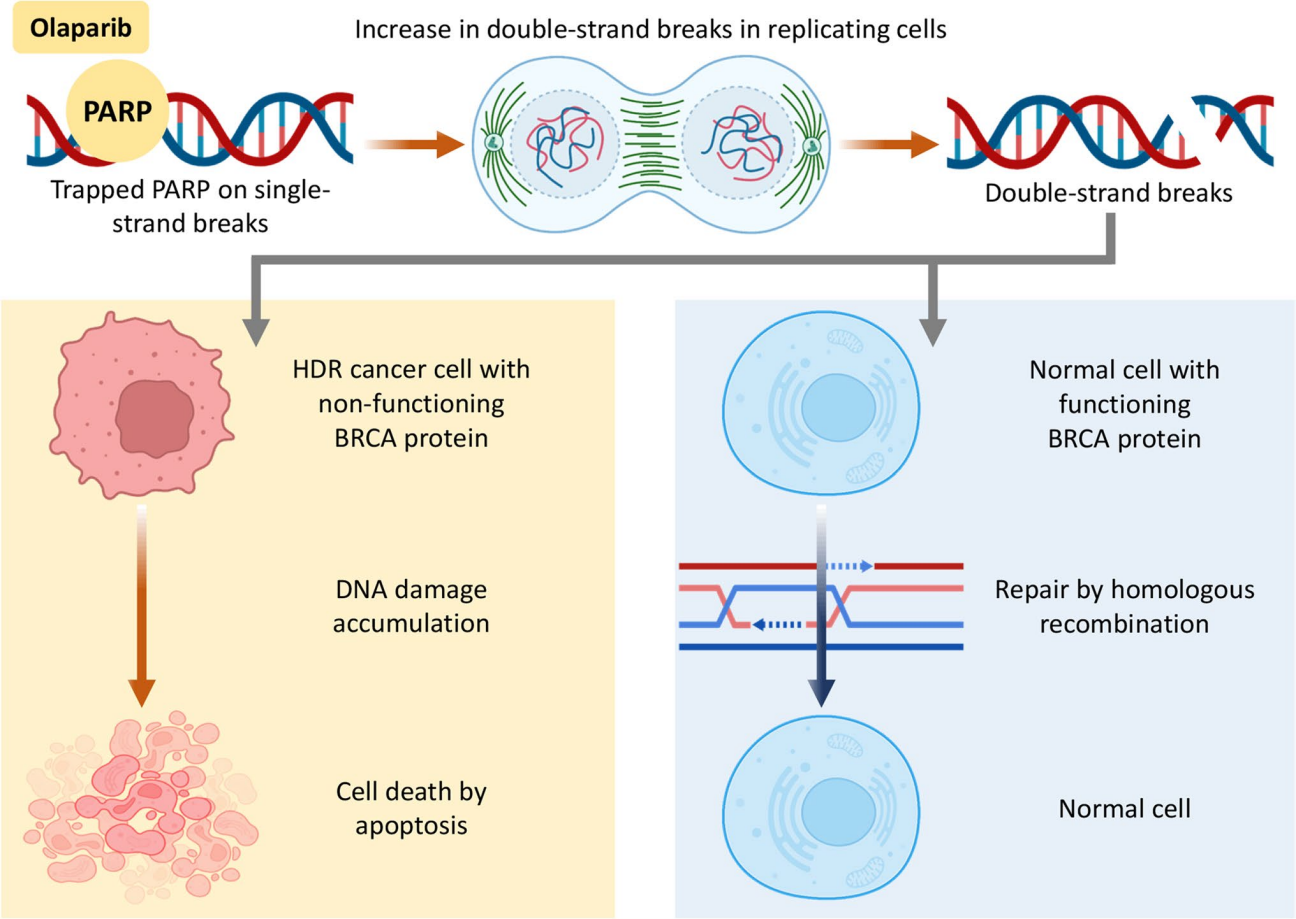
Mechanism of action of Olaparib. Redrawn based on [108].

With more data on the ovarian cancer histology and tumor metagenome, investigations have developed different methods of treatment, such as synthetic lethality. Synthetic lethality is usually involving two genes in cancer cells where the suppression (or mutation) of either one of them does not influence cell viability significantly. However, a simultaneous suppression of both such genes induce cell death. Many cancers (including ovarian malignancies) have already mutated genes involved in vital cellular processes. However, the deficiency in these genes (and mRNAs and proteins) is in most cases compensated by the overexpression of genes coding other similar processes. Consequently, a suppression of such mechanism of compensation by an exogenous treatment will lead to the death of cancer cells called synthetic lethality. At the same time, the absence of the mutation (and therefore active compensation process) in normal noncancerous cells will guard normal cells from the harmful action of the drug. For instance, if cancer cells in ovarian tumor of a patient have a mutant *BRCA1* or *BRCA2* gene, the DNA damage repair pathway in cancer cells is somewhat deficient (Fig. 4). However, another concurrent pathway can mitigate such deficiency and prevent the death of tumor cells. If this compensatory pathway is blocked, the cancer cells will not be able to repair the DNA damage and eventually will die due to the excessive accumulation of the damage [108]. Olaparib, a PARP inhibitor, which has such a mechanism of anticancer action can be used as one of the alternative treatment options for patients with ovarian tumor cells that are lacking the functional *BRCA1* or *BRCA2* genes (Fig. 4).

Another emerging therapy of recurring ovarian cancer is immunotherapy. Immunotherapy is based on the activation of the patient’s own immune system to fight the disease by killing cancer cells and preventing tumor progressing. Immunotherapy can involve targeted antibodies or immunomodulators [109]. Bevacizumab is an example of the targeted antibodies that is used in ovarian cancer patients whether they are newly diagnosed or have relapsed tumors. This monoclonal antibody targets the VEGF/VEGFR pathway inhibiting the growth of blood vessel in the tumor microenvironment [109]. Pembrolizumab is an example of the immunomodulator class of drugs which is used for more specific patients that have an advanced stage cancer with high microsatellite instability, a deficiency in the MMR DNA repair pathway or high tumor mutational burden [109]. This is a checkpoint inhibitor that targets the PD-1/PDL pathway.

Currently developed vaccines against ovarian cancer are mainly based on the use of exogenous or endogenous dendritic cells, cancer testis antigen (CTA), tumor-associated antigens (protein or peptides) in conjunction with adjuvants and recombinant viral vectors expressed cancer antigens [110, 111]. Despite various ongoing clinical trials, there are certain challenges in developing safe and effective therapeutic cancer vaccines in heterogenic and immunosuppressive microenvironment in ovarian cancer. Anticancer vaccines based on the use of nucleic acids (especially mRNA) to generate antigens specific for different cancer attracts a considerable attention of investigators as an alternative to viral vectors and actual proteins/peptides/adjuvants [112].

## Cancer Vaccines in Clinical Trials

Cancer vaccines can be divided into two groups: therapeutic and prophylactic [113]. Since vaccines’ invention and first use by Edward Jenner, they have been developed to train the immune system against specific diseases by ‘learning’ the pathogen’s foreign material and preventing malignancies [114]. Prophylactic vaccines’ mechanism of action is the same as when it was first discovered in late 1700s – they are administered to healthy individuals to prevent disease [113, 115]. Therapeutic vaccines on the other hand are administered to treat the existing disease in a sick individual.

Currently, the number of vaccines available for cancer prevention or treatment is very limited. There are only two prophylactic vaccines available for cancer prevention that have been proven successful: HPV vaccine and hepatitis B vaccine [113, 116–119]. The two FDA-approved prophylactic vaccines against HPV and Hepatitis B are widely in use. HPV infections are one of the most common sexually transmitted diseases and can cause specific carcinomas including but not limited to cervical and anal cancers [120–122]. Although there are more than 80 HPV types identified, some mucosal types prospects oncogenicity and are considered high risk HPV cases [123]. HPV16, 18, 31, 33 belong to this category whereas HPV6 and 11 are categorized as low-risk or non-oncogenic and found in warts [120, 123–125]. Luckily, there are HPV vaccines, Cervarix, Gardasil and Gardasil 9, that strongly protect against up to 9 HPV types which also includes HPV-related cancers [120, 126]. Gardasil 9 is the most comprehensive and the latest HPV vaccine that was approved by the FDA in 2014 [126]. The nine-valent vaccine protects against HPV6, 11, 16, 18, 31, 33, 45, 53, and 58. It has the potential to protect against 90% of cervical cancer cases [126]. Gardasil 9 utilizes virus-like particles that is based on the major capsid protein L1 of papillomavirus [127]. Since the particles used are proteins and do not contain any viral genome, this property makes them non-infectious and non-oncogenic and are considered safer than HPV-attenuated vaccines [120, 128]. HPV vaccines can be produced in various cell types, but Gardasil 9 is produced in yeast cells with aluminum hydroxyphosphate sulfate as the designated adjuvant for a stronger immune response [120]. Just like any other prophylactic vaccine, Gardasil 9 elicits an immune response by initiating the production of neutralizing antibodies and preventing disease.

Hepatitis B, a potentially life-threatening liver infection, is caused by hepatitis B virus (HBV) [129, 130]. HBV is an oncogenic virus that infects hepatocytes, leading to damage in the liver and in some cases can develop into cirrhosis or hepatocellular carcinoma (HCC) [129]. The vaccine developed against HBV is the first anticancer and virus-like-particle based vaccine and is highly effective against all genotypes of HBV [129]. FDA approved HBV vaccines, commercially available such as, Engerix-B and Heplisav-B, both use virus-like particle based on HBsAg which is the surface antigen found on HBV and are produced in yeast cells [131, 132]. Engerix B has 95–100% protective efficacy and HBV vaccines in general have been shown to offer protection for up to 30 years [129, 133].

**Fig. 5:**
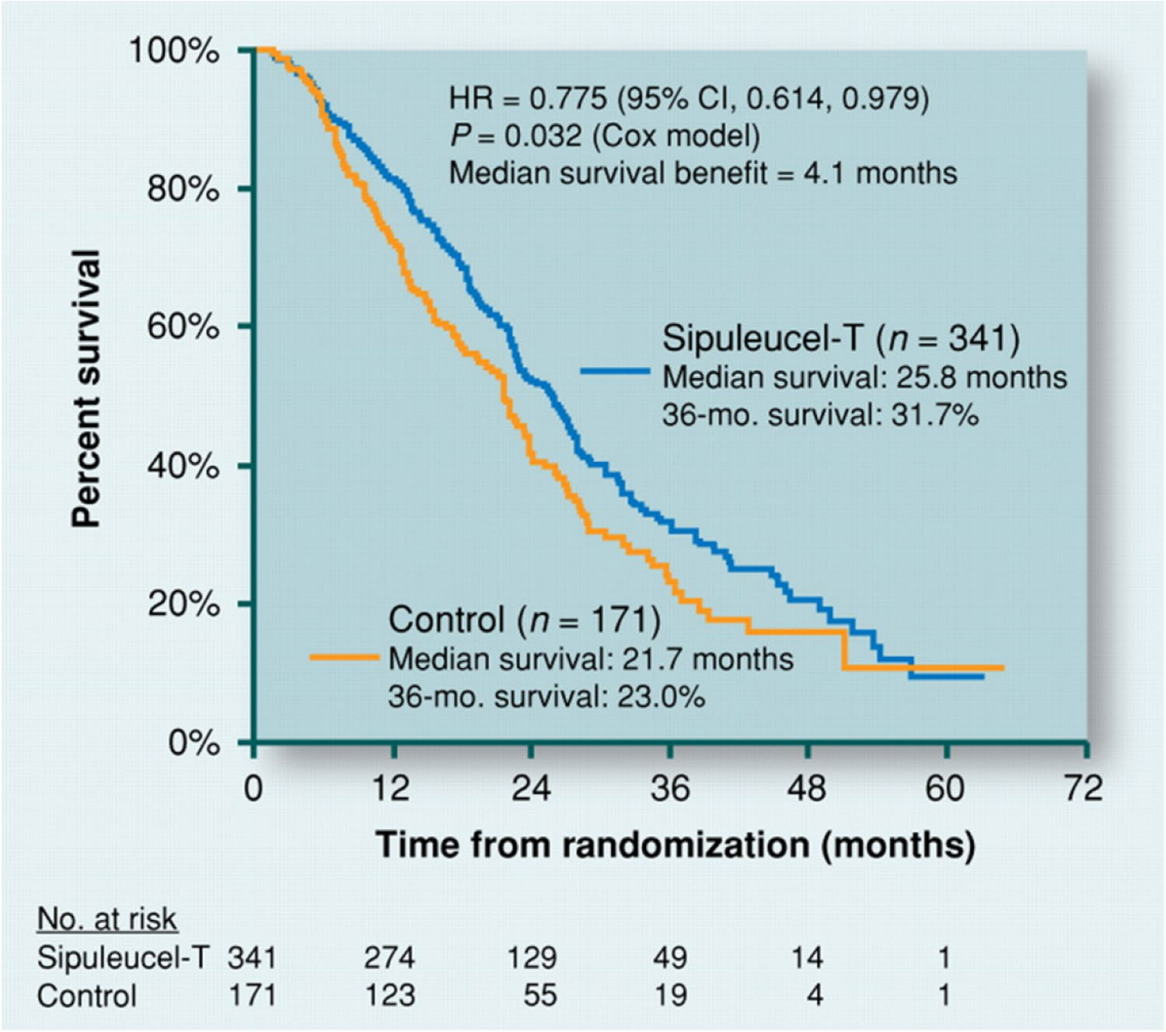
The outcome of efficacy studies with PROVENGE and control. Modified from [117].

Despite extensive clinical trials, FDA so far approved only three therapeutic cancer vaccines. TheraCys ® and TICE, PROVENGE and IMLYGIC [113]. PROVENGE approved in 2010 by the FDA is a therapeutic vaccine used as an immunotherapy agent against metastatic castration resistant prostate cancer [117]. Its mechanism of action is based on alerting the immune system to attack cells that have prostate acid phosphatase (PAP), an antigen presented on the tumor cell surface [117, 134]. PROVENGE activates T cell response in order to kill prostate cancer cells. The vaccine is formulation as a fusion protein called PA2024, which includes recombinant PAP and recombinant granulocyte–macrophage colony-stimulating factor (GM-CSF), a cytokine secreted by various immune cells that helps make more immune cells such as macrophages [117]. In order to receive the treatment, the patient’s blood cells are collected through leukapheresis [135]. The collected cells are then incubated with the PA2024 fusion protein in an attempt to activate antigen presenting cells. Post-incubation, the cells are harvested and transported to a healthcare facility where they can be used for treatment. After the treatment infusion, the antigen presenting cells in the patient’s body proliferate and attack prostate cancer cells carrying the specific antigen [135]. PROVENGE has been used in clinics since the FDA approval and has shown an increase in survival time when compared to control treatments (Fig. 5).

IMLYGIC is a live, attenuated type I herpes simplex virus (HSV-1), that has been genetically engineered in order to express GM-CSF, replicate and lyse tumor cells and enhance immune response by antigen presenting cells [136]. It’s a viral oncolytic therapy against melanoma, the first of its kind, which was approved by FDA in 2015 [136]. HSV-1 is a double stranded DNA virus that has a high lytic potential and is originally isolated from a cold sore that infects the skin [137]. It has been genetically engineered to replicate in selective cell types and propagate lysis by ICP34.5 gene deletion [136]. Further genetic engineering has also achieved by ICP47 gene deletion that enhances MHC class I antigen presentation, and two copies of human GM-CSF gene insertion that promotes immune cell signaling, migration and accumulation [136, 138, 139]. All of these modifications are known to play a major role in the mechanism of action of IMLYGIC, however the exact mechanism of action is now known. Once injected into the patient, the vaccine attacks tumor cells selectively and kills by lysis; enhanced immune cell signaling, and eventual accumulation leads to a better immune response against cancer resulting in better survival rates when compared to control [136].

TheraCys and TICE are both commercial names for the Bacillus Calmette–Guérin (BCG) vaccine, which was first approved and used for the vaccination against tuberculosis [113, 140]. However, it was later approved by the FDA to be used in immunotherapy against early bladder carcinomas in 1990 [113]. It is a live vaccine that uses attenuated *Mycobacterium bovis* when administered via an intravesical route [140]. Its antitumor effect hypothesis was first observed in 1929, however it was approved much later when results from a phase III, double blind, multicenter and randomized study showed promising effects when compared to treatment with doxorubicin [113, 141]. Some studies showed more than 25% decrease of disease progression [142]. Its mechanism of action is still under investigation, although it has been researched for decades. It is thought to exert the antitumor effect similar to the other cancer vaccines in use, which is a combination of direct cytotoxicity, immune cell recruitment, cytokine production and more [143].

There are more than a thousand clinical trials in different stages (to be recruiting, recruiting volunteers at the moment, active but not recruiting, completed and enrolling patients to the trial by invitation) just in the United States alone [144]. Clinical trials involving cancer vaccines include all types of cancers including ovarian, breast, colorectal, lung, pancreas and involves patients with all stages and some examples are show in Table IV.

**Table IV:** Examples of Cancer Vaccines in Clinical Trials

Cancer type	Target	Vaccine type
Extensive-stage small cell lung cancer [145]	Personalized neoantigen	Polyepitope neoantigen DNA vaccine
Non-small cell lung cancer [146, 147]	Her2/neu, CEA, WT1, Mage2, and survivin	Autologous dendritic cell cancer vaccine
Metastatic breast cancer [148]	Her2/neu	Allogeneic gm-CSF-secreting breast cancer vaccine
Late-stage ovarian cancers [149, 150]	TGF-β1 and TGF-β2	Bi-shRNAfurin and GM-CSF augmented autologous tumor cell immunotherapy vaccine
Pancreatic cancer [151]	Mesothelin	Allogenic GM-CSF plasmid-transfected pancreatic tumor cell vaccine
Breast and ovarian cancer [152]	HER-2/neu- or MUC1-derived peptides	Autologous dendritic cells
Ovarian cancer [153]	Mucin 1 (MUC1)	Autologous dendritic cells pulsed with mannan-MUC1 fusion protein (MFP)
Ovarian cancer [154]	Folate receptor	Dendritic cells transfected with mRNA-encoded folate-receptor-alpha
Ovarian cancer [155]	HER-2/neu	Autologous mononuclear cells cultured with a recombinant HER-2/neu
Ovarian cancer clinical trials		

The mechanism of action of therapeutic vaccines is based on tumor associated antigens (TAAs) or tumor specific antigens (TSAs), in which the immune system is trained to destroy cells containing the highly expressed or tumor specific antigens on tumor cells [106]. Most of the previous studies are based on TAAs, are proteins that are expressed in healthy cells and overexpressed cancerous cells, such as HER2 in breast cancer and MUC-1 in adenocarcinoma [114, 156, 157]. Two biggest obstacles in TAA based cancer vaccines are collateral damage and stimulation of low affinity T cells [75, 114]. Since the T cells that bind to self-antigens with a high affinity are eliminated during the T cell selection, the vaccine has to stimulate and activate a “rare” T cell subgroup, which will attack and destroy cells with the overexpressed antigen [75, 114]. However, during this process, activated T cells may damage normal cells as well as cancer cells that are overexpressing the antigens because TAAs are also expressed on normal cells. With vaccines involving TSAs or neoantigens, since the antigens are specifically on tumor cells, they have an immunogenic response in the body naturally [158]. For this reason alone, there is growing interest in cancer vaccines involving neoantigens. There are several phase-I trials for neoantigen based vaccines for advanced melanoma showing promising results [159, 160]. Some obstacles in producing these vaccines are present; the biggest one being the identification of non-synonymous tumor specific mutations to be used and determined as neoantigens [161].

**Fig. 6:**
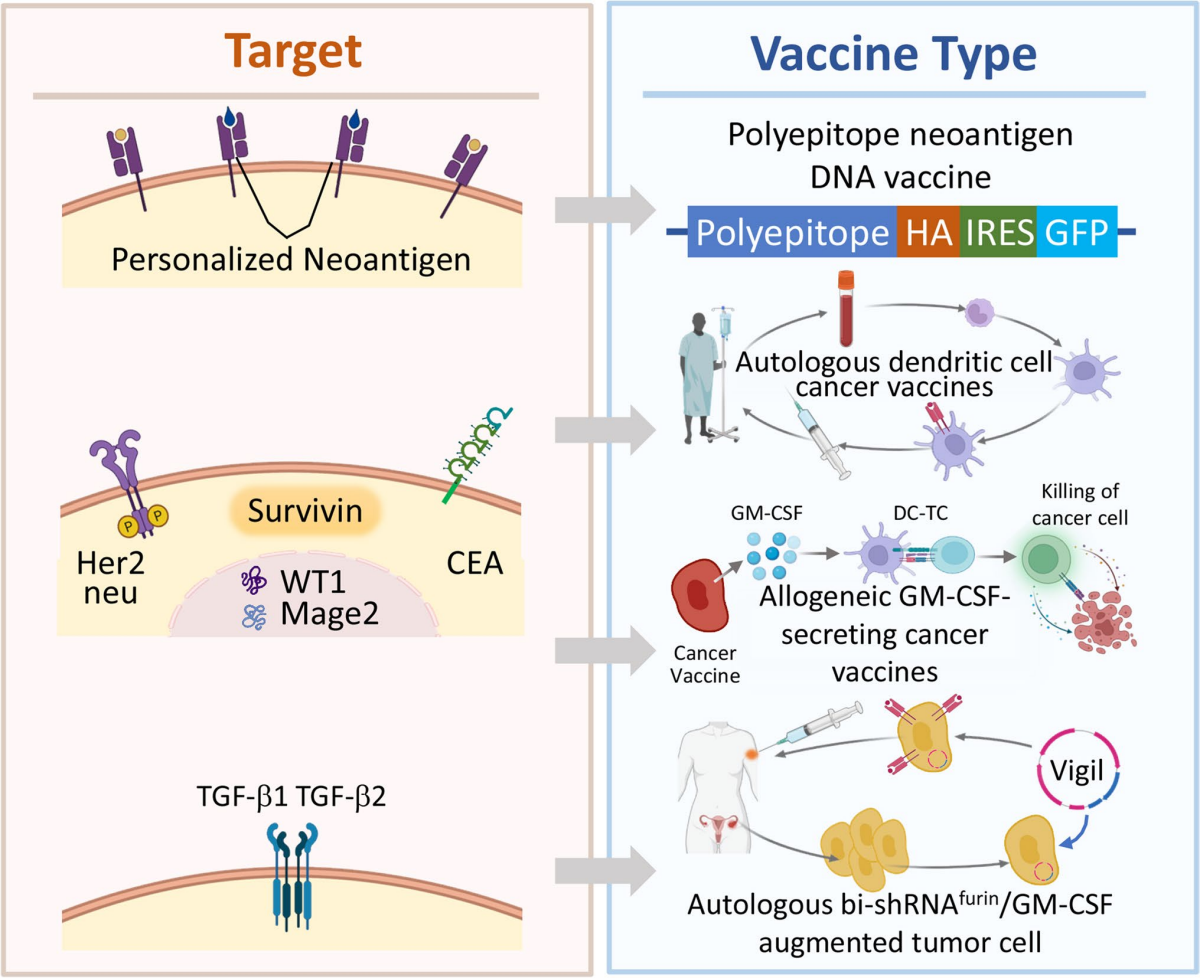
Examples of cancer targets and corresponding vaccines.

It was shown that somatic DNA alterations in cancer cells can produce changes in the sequence of certain membrane proteins/peptides allowing these so-called neoantigens to trigger adaptive immune responses [162]. Because of the mutations in neoantigens, they are not subject to so-called immune tolerance [163]. The main reason of the development of immune tolerance in normal conditions is the prevention of autoimmune diseases. However, the tumor microenvironment induces T-cell tolerance, which in turn promotes uncontrolled tumor growth [164]. Therefore, suppression of this tolerance in cancer patients represents a major challenge in the development of immunologic approaches to cancer treatment. Recently, some major advances in our understanding of tolerance mechanisms in cancer have led to the development of several promising strategies in the development anticancer vaccines. One of the examples of such vaccines (polyepitope neoantigen DNA vaccine) is presented in Fig. 6, upper panel. It includes DNA constructs encoding eight polyepitope model antigens (to target multiple neoantigens), HA-tag and IRES-GFP which were added to allow the detection of polyepitope protein production [162]. It was shown that this optimized polyepitope neoantigen DNA vaccines were capable of inducing antitumor immunity in preclinical models.

Several membrane-bound proteins (*e. g.* human epidermal growth factor receptor 2, HER2/neu, and carcinoembryonic antigen, CEA), cytoplasmic proteins (*e. g.* survivin) or other genes broadly expressed in many tumor types (e. g. Wilms' tumor suppressor gene, *WT1,* Melanoma Antigen Gene, *MAGE*) attracted attention as targets for immunotherapy [162, 165–167]. In particular, these antigens were used in several autologous dendritic cell (DC) cancer vaccines [168]. For the preparation of this type of vaccine, after large scale blood draw and cell separation, dendritic cells are loaded with mentioned above tumor antigens (peptides, proteins, nucleic acids, etc*.*) and matured, often loaded with cytokines, growth factors or TLR ligands dendritic cells are injected back to the patient (Fig. 6, middle panel). Granulocyte–macrophage colony-stimulating factor (GM-CSF)-secreting tumor vaccines represent another variant of cancer vaccine targeted similar antigens [169–171]. It was found that GM-CSF demonstrates a substantial immunostimulatory activity. Injection of irradiated cancer cells stimulated to secrete GM-CSF led to tumor antigen presentation by dendritic cells (DC), activation of CD4 + and CD8 + T-cells (TC) and killing of cancer cells (Fig. 6, middle panel). It was shown in clinical trials that such types of vaccines induced coordinated immune responses with limited toxicity.

Another example of autologous vaccine targeted to ovarian cancer is aimed at specifically reducing expression of furin and downstream TGF-β1 and TGF-β2 (Fig. 6, bottom panel). The vaccine is prepared from the harvested tumor cells which are transfected with the bifunctional shRNA^furin^ DNA sequence plasmid (Vigil) and a granulocyte–macrophage colony-stimulating factor (GM-CSF) DNA sequence [169–172]. Such a transfection stimulates antigen presentation in cancer cells and initiate an adaptive immune response after injection by electroporation back to the same individual. The induction of circulating cytotoxic T lymphocytes capable of destructing autologous tumors after immunization with this type of anticancer vaccine was confirmed in clinical trials.

## mRNA Vaccines

One of the emerging technologies is the use of mRNA vaccines for the treatment or prevention of diseases. When the traditional vaccines usually use a pathogenic protein or an inactivated pathogen particle, mRNA vaccines have the messenger RNA that codes the targeted protein of choice. For instance, nanoparticles containing mRNAs that encode several viral proteins, their different domains or peptides (*i.e.*spike, membrane, envelope, etc*.*) are used as a vaccine against SARS-CoV-2 [173, 174]. Such mRNA-based vaccine demonstrated a considerable success for the population of the world. There are many advantages in the mRNA pharmacology due to its nature. mRNA is a non-infectious and non-integrating piece of genetic materials that doesn’t impose any genetic risks and should be tolerable [119]. It also has the potential to stimulate both CD4, CD8 cells and B cell mediated humoral immune response [119]. Despite a relatively high cost of mRNA synthesis, they are also cheaper, highly potent and have the potential to be faster to develop when compared with conventional vaccines [175].

**Fig. 7:**
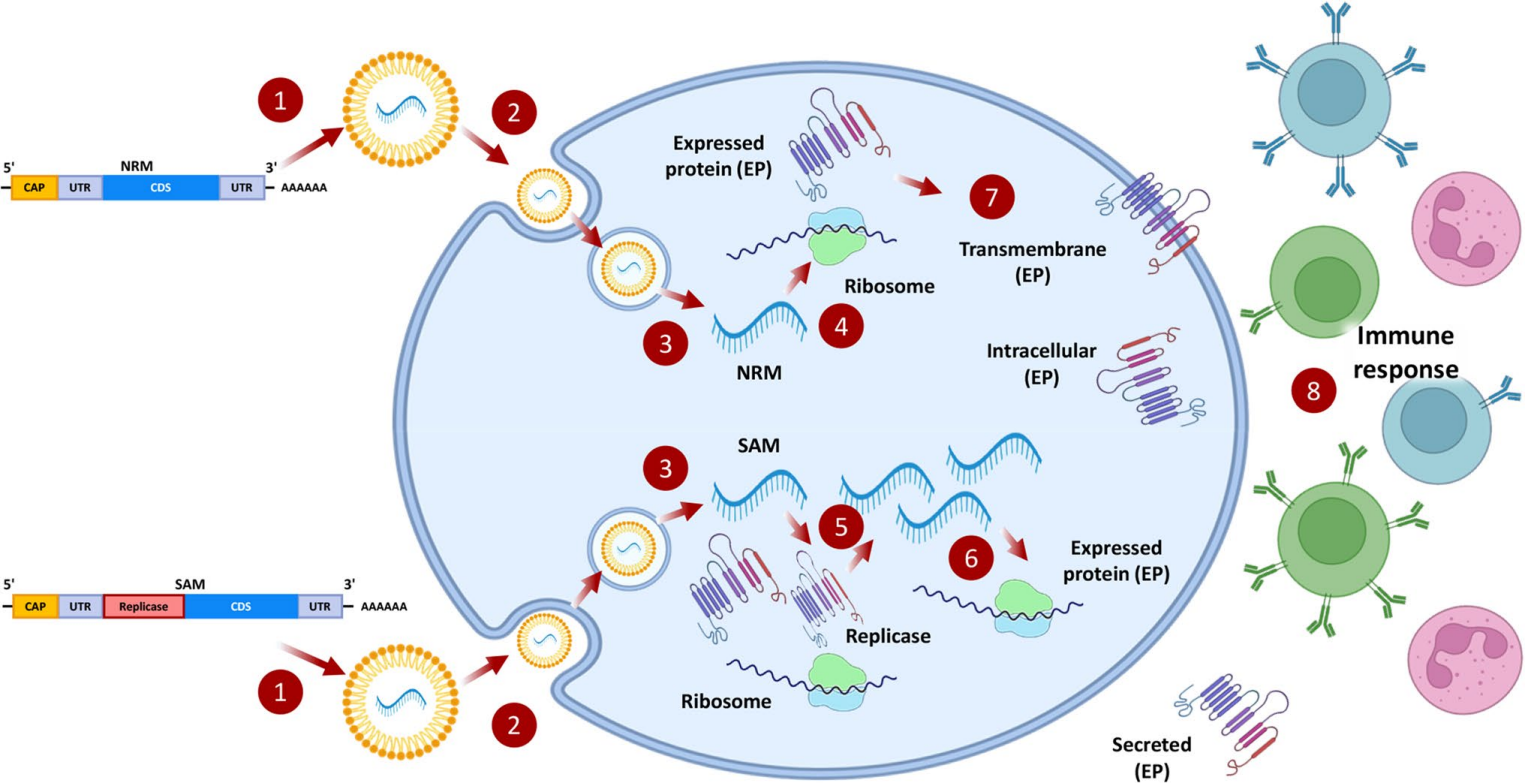
Mechanism of action of mRNA vaccines. 1—Non-replicating mRNA (NRM) and self-amplifying mRNA (SAM) constructs are formulated with lipid nanoparticles which encapsulate the mRNA, protect the nucleic acid from degradation and facilitates its cellular internalization. 2—The cellular uptake of mRNA-containing nanoparticles usually occurs by endocytosis. 3- mRNA is released into the cytosol by endosomal escape. 4—NRM constructs are translated by ribosomes into expressed proteins, which undergo post-translational modifications. 5—SAM constructs can also be translated by ribosomes to produce the machinery required for replication in the self-amplification process. 6—Self-amplified mRNA constructs can be translated by ribosomes to produce the protein. 7—The expressed proteins are secreted outside of the cell. 8—The immune system detects the protein and activates developing an immunological memory which allows the immune system to respond rapidly and effectively to pathogen. Redrawn from [176].

The mechanism of action of a mRNA vaccine is to transfer the transcript of interest that will encode one or more immunogens into the host where the cells will produce these proteins to locate it intracellularly, within the membrane or to secrete it [176, 177]. There are two major types of constructs being evaluated (Fig. 7): non-replicating mRNA (NRM) and self-amplifying mRNA (SAM). These types have similar modes of action and constructs. NRM and SAM constructs have an open reading frame, a cap structure, 5′ and 3′ untranslated regions (UTRs), and a 3′ poly-A tail [176, 177]. The big difference between the two is that SAM is involved in a genetic replication machinery that is derived from positive-stranded mRNA viruses [176, 178, 179]. After the delivery of mRNA inside the cancer cell (e. g. by lipid nanoparticles as shown in Fig. 7), mRNA is released into the cytosol where it is processed by ribosomes to synthesize corresponding protein [176]. The difference between the two constructs is that a protein synthesized from SAM requires post-translational modifications, but NRM does not [176]. Once the expressed protein is ready it will be displayed at the appropriate location. Even though there are more cellular pathways involved in the SAM processing, this type of vaccine has an advantage of being self-amplifying and replicating on its own and producing antibodies without a booster [119].

The number of research efforts already have been dedicated to different types of nucleic acids for developing of cancer vaccines and various approaches to personalized medicine. One study focused on developing a personalized cancer vaccine using the genomic background of cancer cells within each individual patient receiving treatment [180]. Several other approaches are investigated in our laboratory on generating nanoparticles with siRNAs targeted specific mechanisms of cancer cell resistance to chemotherapy overexpressed in ovarian cancer cells obtained from resected tumor tissues of each individual patient with an advanced stage of the disease [181–189].

Based on the mentioned above reasons, the tumor biomarker CA125 can potentially be used for the development of mRNA vaccines against epithelial ovarian cancer, specifically HGSC. Only few manuscripts dedicated to a treatment of ovarian cancer using dendritic cells and short hairpin RNA have been published. A considerable success in the upregulation of immune cells by short hairpin RNA targeting the *Mucin16* (*MUC16*) gene administered in human cancer cell lines [88]. Therefore, there is an urgent need for a new treatment option for the most common and the most lethal ovarian cancers. Based on the aforementioned, it seems that an mRNA construct encoding CA125 protein, which is upregulated and overexpressed in HGSC tumor cells, delivered by tumor targeted nanoparticles might represent an effective alternative approach for treating of ovarian cancer. In this approach, the use of SAM constructs will be advantageous due to their self-amplifying nature, which erases the need for weekly treatments usually required by the patients receiving conventional vaccines.

## Delivery of mRNA Vaccines

**Fig. 8:**
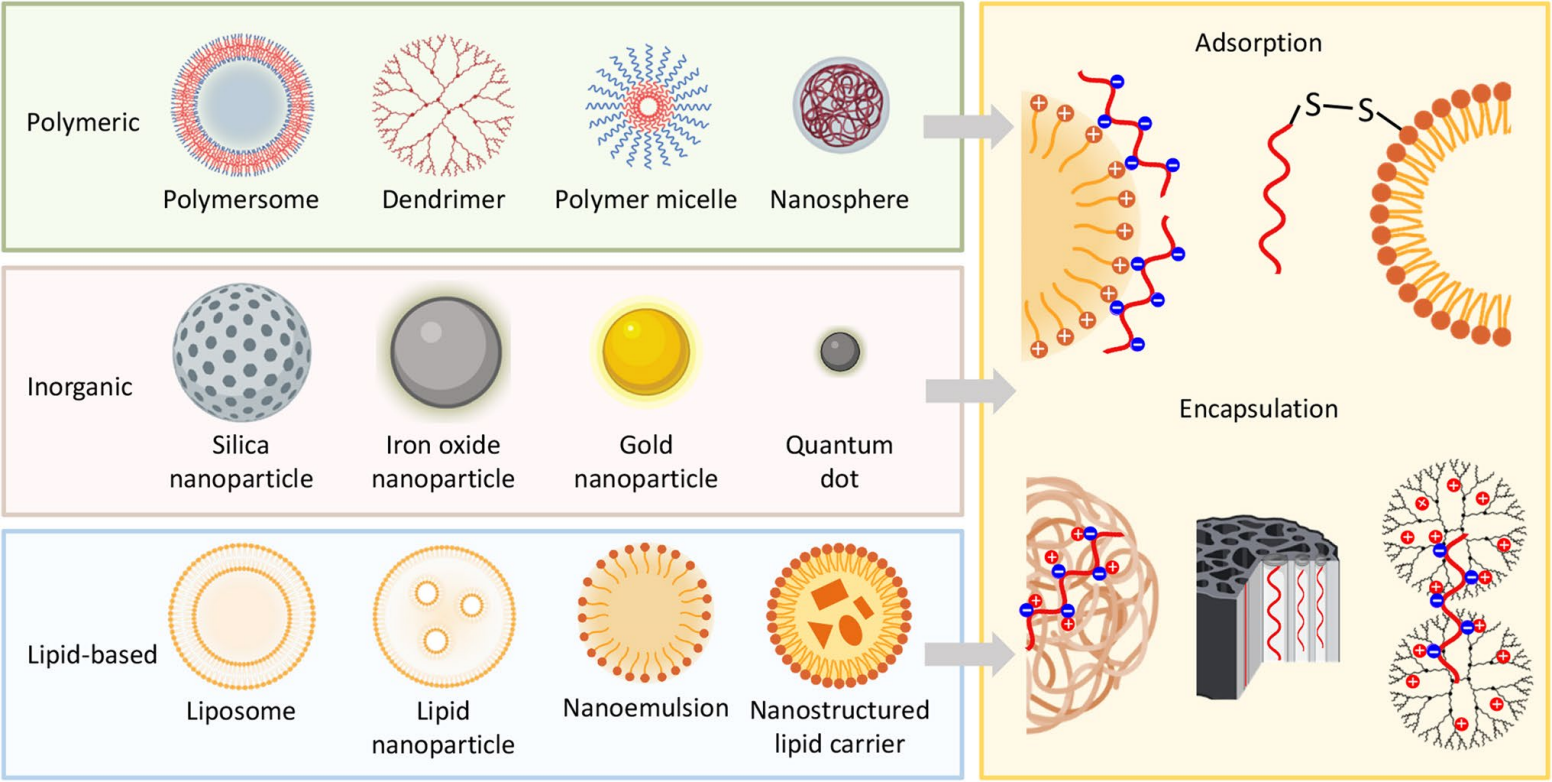
Examples of nanoparticle types (left panel) and various approaches (right panel) for the RNA delivery.

It is well known that nucleic acids (especially RNAs) are pretty unstable and cannot be delivered in their naked form and therefore require special nanotechnology-based delivery systems [173, 177, 183, 185, 186, 190]. Such delivery systems solve several tasks. First, they protect nucleic acids from the degradation during their journey inside human body. Second, the delivery system is easily internalized by cells releasing mRNA into the cytoplasm. In addition, the delivery system may be targeted to the specific cells by a targeting moiety or other methods [174, 188]. Several types of nanoparticles can be used to deliver nucleic acids including mRNA-based vaccines. Figure 8 (left panel) gives examples of nanoparticles suitable for this purpose. Three main types of nanoparticles – polymeric, inorganic and lipid-based, can potentially be used for the delivery of mRNA vaccines. Many types of polymers—PCL—poly(ε-caprolactone); PEG—poly(ethylene glycol); PLA – poly(lactic acid; PLGA – poly(lactide-co-glycolide); HPMA – N-(2-Hydroxypropyl) methacrylamide and many others—are being used for preparation of nanoparticles of various architecture. The most common types of them are presented in the upper panel of Fig. 8 and included various assemblies with relatively simple spheric structures (polymersomes, micelles, nanospheres, etc.) or highly defined constructs (*e.g*. dendrimers). Nanoparticles for drug delivery can also be fabricated from different non-organic materials (Fig. 8, middle panel) including metals (*e.g.* iron, gold), silica or semiconductors (*e.g.* quantum dots). Different lipid compositions are widely employed for the preparation of various nanoparticles capable of delivering hydro- and lipophilic compounds as well as nucleic acids (Fig. 8, bottom panel). Lipid-based nanoparticles are currently the most frequently used vehicles for mRNA vaccines [119, 173, 176, 185]. Two major approaches are utilized for the incorporation of RNA (as well as other nucleic acids) into a complex delivery system: (1) adsorption, when a nucleic acid is coupled on the surface of a nanoparticle, and (2) encapsulation, when it placed inside a single nanoparticle or several nanocarriers (Fig. 8, left panel). A nucleic acid with negative charge in normal pH conditions can be bound to the surface of positively charged (cationic) nanoparticles. Alternatively, a chemical conjugation (e. g. via a disulfide bond) can also be used to connect chemically modified RNA to a nanoparticle. Encapsulation of mRNA into nanoparticles can be achieved by several approaches. A negatively charged nucleic acid can be incorporated into a nanoparticle (e. g. nanosphere) or “covered” by several nanoparticles with (e. g. dendrimers) with internal positive charge. A nucleic acid molecule can also be encapsulated into sealed internal pores of certain types of nanocarriers (e. g. mesoporous silica nanoparticles). In addition, nanoparticle-mRNA complexes can be functionalized with targeting moieties directing them specifically to the targeted cells, e. g. cancer cells. The recent example vaccines with encapsulated mRNA are COVID-19 vaccines developed by Pfizer or Moderna [173, 191].

Based on the search of U.S. National Library of Medicine on October 2022 more than 100 clinical studies on vaccines against ovarian cancer were registered and 22 of them are currently active or recruiting patients (Table V) [192]. However, only two of them are use nucleic acid-based vaccines testing liposomal form of mRNA encoding three tumor-associated antigens (TAAs) specific for ovarian cancer and plasmid-based DNA vaccine encoding the ICD of HER2 (NCT Numbers: NCT00436254 and NCT04163094, respectively).

**Table V:** Ongoing Clinical Trials On Vaccines Against Ovarian Cancer [192]

#	NCT Number	Title	Status*	Phase(s)
1	NCT04163094	Ovarian Cancer Treatment with a Liposome Formulated mRNA Vaccine in Combination With (Neo-)Adjuvant Chemotherapy	A, NR	1
2	NCT04739527	Phase 1 Study to Evaluate the Safety, Feasibility and Immunogenicity of an Allogeneic, Cell-based Vaccine (DCP-001) in High Grade Serous Ovarian Cancer Patients After Primary Treatment	R	1
3	NCT01309230	Trial of Adjuvant FANGâ„¢ Vaccine for High Risk Stage III/IV Ovarian Cancer	A, NR	2
4	NCT00703105	Ovarian Dendritic Cell Vaccine Trial	R	2
5	NCT02737787	A Phase I Study of WT1 or NY-ESO-1 Vaccine and Nivolumab for Recurrent Ovarian Cancer	A, NR	1
6	NCT00799110	Vaccination of Patients with Ovarian Cancer With Dendritic Cell/Tumor Fusions With Granulocyte Macrophage Colony-stimulating Factor (GM-CSF) and Imiquimod	A, NR	2
7	NCT05479045	A Combination Therapy Strategy to Prevent Anti-PD-1 Therapy Resistance in Metastatic Ovarian Cancer Patients	NYR	2
8	NCT05270720	Dendritic Cell Vaccination with Standard Postoperative Chemotherapy for the Treatment of Adult Ovarian Cancer	NYR	1
9	NCT04024878	NeoVax With Nivolumab in Patients with Ovarian Cancer	R	1
10	NCT00194714	Vaccine Therapy in Treating Patients with Stage IV HLA-A2 and HER2 Positive Breast or Ovarian Cancer Receiving Trastuzumab	A, NR	1|2
11	NCT04713514	OSE2101 Alone or in Combination with Pembrolizumab *vs* BSC in Patient with Platinum-sensitive Recurrent OC	R	2
12	NCT05104515	First-in-human Study of OVM-200 as a Therapeutic Cancer Vaccine	R	1
13	NCT02785250	Study of DPX-Survivac Therapy in Patients with Recurrent Ovarian Cancer	A, NR	1|2
14	NCT03735589	Specialized Immune Cells (nCTLs) and a Vaccine (Alpha-type-1 Polarized Dendritic Cells) in Treating Patients with Stage II-IV Ovarian, Fallopian Tube, or Primary Peritoneal Cancer	NYR	1|2
15	NCT02111941	Vaccine Therapy in Treating Patients with Stage IIIC-IV Ovarian Epithelial, Fallopian Tube, or Primary Peritoneal Cavity Cancer Following Surgery and Chemotherapy	A, NR	1
16	NCT03206047	Atezolizumab, Guadecitabine, and CDX-1401 Vaccine in Treating Patients with Recurrent Ovarian, Fallopian Tube, or Primary Peritoneal Cancer	A, NR	1|2
17	NCT00436254	Vaccine Therapy with Sargramostim (GM-CSF) in Treating Patients with Her-2 Positive Stage III-IV Breast Cancer or Ovarian Cancer	A, NR	1
18	NCT03318900	T-Cell Infusion, Aldesleukin, and Utomilumab in Treating Patients with Recurrent Ovarian Cancer	A, NR	1
19	NCT03113487	P53MVA and Pembrolizumab in Treating Patients with Recurrent Ovarian, Primary Peritoneal, or Fallopian Tube Cancer	A, NR	2
20	NCT03029403	Phase 2 Study of Pembrolizumab, DPX-Survivac Vaccine and Cyclophosphamide in Advanced Ovarian, Primary Peritoneal or Fallopian Tube Cancer	R	2
21	NCT01849874	A Study of MEK162 *vs*. Physician's Choice Chemotherapy in Patients with Low-grade Serous Ovarian, Fallopian Tube or Peritoneal Cancer	A, NR	3
22	NCT03311334	A Study of DSP-7888 Dosing Emulsion in Combination with Immune Checkpoint Inhibitors in Adult Patients with Advanced Solid Tumors	A, NR	1|2

^*^ Abbreviations: A – Active, NR – Not recruiting; NYR – Not yet recruiting

## Future Directions

The major advantage of nucleic-based vaccines in general is the ability to stimulate an immune response against disease-causing pathogen without a physical presence of such a pathogen. They have a distinct advantage over traditional vaccines that use an entire bacterium, virus, or other microorganism. In contrast, nucleic-based vaccines use just a genetic material which encodes a protein specific to the pathogen in order to initiate the immune response without introducing an entire virus or its protein(s). As a result, the immune response and immune memory is formed without the risk of acquiring the disease. Such the advantage can be realized when the following major requirements are fulfilled. First, the selected pathogen protein encoded by the vaccine nucleic acid should be highly specific to the pathogen and immunogenic meaning that this protein should provoke a stable immune response. In case of viruses, the selection of such a protein is relatively straightforward. However, for the anticancer vaccines, such a selection is significantly more difficult. On the one hand, the selected protein should be highly specific to the targeted cancer. On the other hand, it must provide initiate a strong immune response by present a major histocompatibility complex or antigen on the cell surface. The accurate selection of a targeted protein in order to create a strong anticancer vaccine on our opinion currently represents a major challenge in the development of effective anticancer vaccines. The further selection of a nucleic acid encoding an entire protein or its essential peptide represent a substantially easy task taking into account modern molecular biology techniques. Second, to effectively generate a protein antigen, a nucleic acid must be effectively delivered inside a cell nucleus (in case of DNA) or cytoplasm (for RNA). In this sense, RNA is preferred for anticancer (as well other types) vaccines, because it acts in the cytoplasm (and therefore should not be delivered inside the cell nucleus) and cannot be inherited by daughter cells. Since a naked nucleic acid hardly penetrate cellular and nuclear membrane, it requires an effective delivery system. Moreover, such transport system not only should deliver and release DNA or RNA through the cellular membrane only in immune competent cells leaving other cells of the body intact. This cell-specific delivery of nucleic acids represents a major obstacle to the development effective and targeted delivery of anticancer vaccines. Third, there are some problems in the production and storage of anticancer (as well as other types) of nucleic acid-based vaccines that need to be solved in order to provide effective immunization of population at risk. On our opinion, these major challenges should be addressed and represent an immediate further direction in the development and clinical applications of anticancer vaccines.
